# High-Resolution Melting Analysis to Detect Antimicrobial Resistance Determinants in South African *Neisseria gonorrhoeae* Clinical Isolates and Specimens

**DOI:** 10.1155/2022/9094328

**Published:** 2022-01-18

**Authors:** Nireshni Mitchev, Ravesh Singh, Veron Ramsuran, Arshad Ismail, Mushal Allam, Stanford Kwenda, Florah Mnyameni, Nigel Garrett, Khine Swe Swe-Han, Abraham J. Niehaus, Koleka P. Mlisana

**Affiliations:** ^1^School of Laboratory Medicine and Medical Sciences, University of KwaZulu Natal (UKZN), Durban, South Africa; ^2^National Health Laboratory Service, Durban, South Africa; ^3^Sequencing Core Facility, National Institute for Communicable Diseases, National Health Laboratory Service, Johannesburg, South Africa; ^4^Department of Genetics and Genomics, College of Medicine and Health Sciences, United Arab Emirates University, Al Ain, UAE; ^5^Centre for the AIDS Programme of Research in South Africa, Durban, South Africa; ^6^School of Nursing and Public Health, UKZN, Durban, South Africa; ^7^National Health Laboratory Service, Johannesburg, South Africa

## Abstract

**Background:**

Antimicrobial resistance is limiting treatment options for *Neisseria gonorrhoeae* infections. To aid or replace culture and the syndromic management approach, molecular assays are required for antimicrobial susceptibility testing to guide appropriate and rapid treatment.

**Objective:**

We aimed to detect single-nucleotide polymorphisms and plasmids associated with antimicrobial resistance from *N. gonorrhoeae* isolates from a clinic population in South Africa, using real-time PCR as a rapid test for AMR detection.

**Methods:**

*N. gonorrhoeae* isolates, from female and male patients presenting for care at a sexually transmitted infections clinic in Durban, South Africa, were analysed using phenotypic and genotypic methods for identification and antibiotic susceptibility testing (AST). Real-time PCR and high-resolution melting analysis were used to detect *porA* pseudogene (species-specific marker) and resistance-associated targets. Whole-genome sequencing was used as the gold standard for the presence of point mutations.

**Results:**

The real-time *porA* pseudogene assay identified all *N. gonorrhoeae*-positive isolates and specimens. Concordance between molecular detection (real-time PCR and HRM) and resistance phenotype was ≥92% for *bla*_TEM_ (HLR penicillin), rpsJ_V57M (tetracycline), *tetM* (tetracycline), and gyrA_S91F (ciprofloxacin). Resistance determinants 16SrRNA_C1192U (spectinomycin), mtrR_G45D (azithromycin), and penA_D545S, penA_mosaic (cefixime/ceftriaxone) correlated with the WHO control isolates.

**Conclusions:**

Eight resistance-associated targets correlated with phenotypic culture results. The *porA* pseudogene reliably detected *N. gonorrhoeae*. Larger cohorts are required to validate the utility of these targets as a convenient culture-free diagnostic tool, to guide STI management in a South African population.

## 1. Introduction

Sexually transmitted infections (STIs) are a global public health concern, with an annual estimate of 87 million new cases of *Neisseria gonorrhoeae* infection [1]. Globally, *N. gonorrhoeae* has developed resistance to most antibiotics, including third-generation cephalosporins, cefixime and ceftriaxone. The World Health Organization (WHO) call to end the STI epidemic as a public health concern emphasizes a need to strengthen technologies and improve knowledge on the prevalence, resistance patterns, and public health interventions to combat STIs, especially in low- and middle-income countries [2].

For *N. gonorrhoeae*, the sensitivity of microscopy for diagnosis is 90–95% in men and 50–70% for presumptive diagnosis in women [3]. The sensitivity of culture ranges from 85–95% in patients with recent and symptomatic infection to as low as 50% in asymptomatic patients [4, 5]. A challenge to STI management is delayed or ineffective treatment initiation whilst awaiting laboratory diagnosis (48–72 hours), which could lead to patient loss to follow-up, [6, 7] and increased risk of infection transmission to sexual partners. In well-resourced settings, sensitive and rapid nucleic acid amplification tests (NAATs) have largely replaced phenotypic identification of *N. gonorrhoeae* [5]. Although NAATs are effective in diagnosis at POC, most settings treat patients empirically at the first visit.

Syndromic and empiric treatment contributes to the development of resistance to currently recommended drugs in many parts of the world [8–10]. *N. gonorrhoeae* mechanisms of resistance have been well documented [11–13]. These include plasmid-mediated resistance to penicillin (*bla*_TEM_) and tetracycline (*tetM*) and chromosomally mediated resistance to penicillin, tetracycline, spectinomycin, fluoroquinolones, azithromycin and cephalosporins [11]. Resistance to penicillin and extended-spectrum cephalosporins has been associated with modifications and recombination within *penA, porB,* and *ponA* [14]. The mutation L421P in *ponA* reduces the rate of acylation with penicillin [15]. Mutations in *mtrR*, as well as its promoter region, can cause overexpression of the mtrCDE efflux pump which has been associated with resistance to penicillin, tetracycline, cefixime, ceftriaxone, and azithromycin [16, 17]. Mutations in *porB* which encode porinB, reduces the porin permeability. The *pilQ* gene encodes for pore formation in the outer membrane, mutations in which result in reduced antibiotic influx [11, 13, 18, 19]. Resistance to tetracycline has been associated with mutations in *rpsJ*, *mtrR*, and *porB* [16]. The *rpsJ* mutation V57M alters the binding site, thus reducing the binding affinity of tetracycline for the ribosome [11]. Resistance to ciprofloxacin is due to mutations in *gyrA* and *parC* [16]. High-level resistance to spectinomycin is due to the mutation C1192U in 16S rRNA by reducing antibiotic target affinity [20]. Resistance to azithromycin is often due to mutations in 23S rRNA, namely, C2611T (low-level resistance) or A2059 (high-level resistance) [11, 20].

Molecular antimicrobial resistance testing is imperative for the control of *N. gonorrhoeae*, to facilitate antibiotic stewardship, to expedite appropriate treatment of patients, and to conserve the effectiveness of the current treatment regimen [21]. A recent study projected that continued empiric treatment without antimicrobial susceptibility testing at the POC would result in >5% of *N. gonorrhoeae* isolates being resistant to both azithromycin and ceftriaxone within 15 years, but the use of a molecular assay could delay the emergence of resistance by 5 years [22, 23]. Currently, the only commercially available genotypic resistance testing assay for *N. gonorrhoeae* is from SpeeDX (Australia), which determines resistance to ciprofloxacin by detecting the gyrA_S91F mutation. However, due to the high prevalence of resistance to ciprofloxacin in South Africa and other African countries [21], additional assays to detect genotypic resistance to other drugs are necessary.

Whole-genome sequencing and a variety of bioinformatics tools are widely used to predict AMR and identify novel mutations in *N. gonorrhoeae*. These tools include Resistance Gene Identifier (RGI), Basic Local Alignment Search Tool (BLAST), Rapid Annotation Using Subsystems Technology (RAST), Antibiotic Resistance Gene-ANNOTation (ARGANNOT), ResFinder, ARIBA, ABRicate, ResFinder, PointFinder, and NG-STAR [24]. However, the cost of this technology is high, especially in low–middle-income-countries (LMIC).

Real-time polymerase chain reaction (real-time PCR) combines amplification and detection of gene targets in a single assay. Advantages include rapid detection, the ability to be implemented in high throughput settings [25], and cost effectiveness [26]. The *porA* pseudogene is a *N. gonorrhoeae* species-specific marker [27–29] and is highly conserved across a diverse range of *N. gonorrhoeae* strains making it a useful target for identification [27, 28, 30, 31].

High-resolution melt (HRM) analysis is a post-PCR analysis method, which amplifies gene targets in the presence of a fluorescent reporter dye; the increasing temperature gradient denatures the DNA and releases the dye, which then undergoes a conformational change that reduces the ﬂuorescence. An HRM instrument records the ﬂuorescence fluctuation and produces a melt curve of the target gene, which can be used to compare the similarity of PCR products [32]. HRM is so sensitive that it can detect a single-nucleotide polymorphism (SNP) [30]. This technology is rapid and cost effective [33], a cheaper alternative to sequencing [32, 34], and can be employed to detect SNPs associated with antibiotic resistance [30, 35, 36].

Developing and evaluating new technologies for AMR prediction directly from patient specimens, to be used at the POC or near-patient, is currently a priority to conserve current and future antimicrobials [18]. Some resistance determinants may work on their own in increasing resistance in particular drugs. However, in some cases, a single determinant may only alter the minimum inhibitory concentration (MIC) slightly or not at all [11]. In our study, we correlated known AMR determinants with phenotypic AST (gold standard) data from our local isolates to determine which mutations in our population result in a resistant phenotype. Using real-time PCR and HRM technologies, we detected genes and mutations associated with antimicrobial resistance to penicillin, tetracycline, spectinomycin, ciprofloxacin, azithromycin, ceftriaxone, and cefixime in our local population.

## 2. Materials and Methods

### 2.1. Study Population and Sample Collection

Genital samples were collected from symptomatic male and female patients attending a large urban STI clinic in Durban, South Africa, as part of two clinical studies. Female patients (May 2016 –January 2017), aged 18–40 years, consented to vaginal swab collection (Eswab®, Copan, Brescia, Italy), as reported previously [37], and male patients (June–August 2015), aged 19–60 years, consented to urethral Eswab® collection. A total of 22 *N. gonorrhoeae*-positive specimens were included in this study. Ethics was approved for this study by the Biomedical Research Ethics Committee of the University of KwaZulu-Natal, BREC97/2019.

### 2.2. Isolation and Identification of *Neisseria gonorrhoeae*

Phenotypic identification included bright-field (Gram stain) microscopy and culture. Swabs were inoculated onto New York City and chocolate agar media and incubated for 24–48 hours at 37°C in a 5% CO_2_ incubator. Suspected *N. gonorrhoeae* colonies were confirmed using the Rapid Oxidase test and Phadebact® Monoclonal GC test (Pharmacia, Sweden). STI screening, directly from patient specimens, was performed using NAATs, Anyplex™ II STI-7 Detection (Seegene, Seoul, Korea), and Xpert^®^ (Cepheid, CA, USA). Isolates were stored in the laboratory repository for future phenotypic and genotypic analysis.

### 2.3. Antimicrobial Susceptibility Testing

*N. gonorrhoeae* isolates from vaginal and urethral specimens were revived on nonselective Thayer Martin (antibiotic supplement excluded) and chocolate agar media for 18–24 hours in a 37°C 5% CO_2_ incubator. WHO *N. gonorrhoeae* control strains (F, G, K, L, M, N, O, and P) [38] and ATCC strain 49226 were used in this study. AST using Etest® (bioMérieux, Marcy l'Etoile, France) was performed for penicillin, tetracycline, spectinomycin, ciprofloxacin, azithromycin, ceftriaxone, and cefixime, as per the manufacturer's guidelines, using GC agar base medium supplemented with 1% Vitox [39, 40]. The European Committee on Antimicrobial Susceptibility Testing (EUCAST) guidelines [41] were used to interpret MICs.

### 2.4. DNA Extraction

Genomic DNA was isolated from *N. gonorrhoeae* isolates and control strains (WHO F, L, O, G, M, P, N, and K) [38] using the Quick-DNA™ Miniprep Plus Kit (Zymo Research, CA, USA) as per the manufacturer's instruction. DNA was extracted from patient specimens as follows: swabs were vortexed for 30 s while inside their Eswab® transport tubes, whereafter 200 *µ*L of the suspension was added to a 1.5 mL Eppendorf tube, and the Quick-DNA™ Miniprep Plus Kit (Zymo Research, CA, USA) was used as per the manufacturer's instruction.

### 2.5. Real-Time PCR for porA, bla_TEM_, and tetM

Real-time PCR was performed on the Quant Studio 5 (ThermoFisher, CA, USA), to detect *porA* for identification of *N. gonorrhoeae* and plasmids *bla*_TEM_ and *tetM*, which confers resistance to penicillin and tetracycline, respectively.

### 2.6. High-Resolution Melt Analysis

We screened *penA*, *porB, ponA*, *gyrA*, *parC*, *mtrR*, *rpsJ*, and 16s rRNA for the presence of SNPs associated with AMR to penicillin, tetracycline, spectinomycin, ciprofloxacin, azithromycin, cefixime, and ceftriaxone, as previously described [12]. Briefly, PCR was employed to amplify the chromosomal genes (Table 1) on the SimpliAmp instrument (Life Technologies, CA, USA), using the KAPA HiFi PCR kit (KAPA Biosystems, MA, USA) as per the manufacturer's instructions. Cycling conditions were as follows for the first-round primers: 95°C for 3 minutes, 30 cycles of 98°C for 30 seconds, 55°C for 30 seconds, and 72°C for 30 seconds a final extension reaction at 72°C for 7 minutes. Samples were run on a 2% agarose gel to confirm PCR amplification. The PCR product was diluted (1 : 4000) and used as the template for HRM assays. To detect SNPs that associate with phenotypic resistance, we used HRM technology, performed on the Quant Studio 5 (ThermoFisher, CA, USA). Each HRM reaction (20 *μ*l total volume) consisted of 10 *μ*l MeltDoctor™ HRM Master Mix (Applied Biosystems, CA, USA), 1.2 *μ*l of each primer (0.3 pmol/*μ*l), 1 *μ*l DNA template, and nuclease-free water. Reactions were performed on the Quant Studio 5 (ThermoFisher, CA, USA) as follows: initial denaturation at 95°C for 10 min, followed by 35 cycles at 95°C for 15 s and 60°C for 1 min. HRM analysis was performed as follows: an initial holding step for 1 min at 60°C, followed by a slow temperature increase at a rate of 0.075°C/s to 95°C with continuous fluorescence signal collection. The results were analysed using High-Resolution Melt Software v3.1 (Applied Biosystems™).

### 2.7. Whole-Genome Sequencing and Assembly

DNA was extracted from isolates using the PureLink™ Microbiome DNA Purification Kit (ThermoFisher Scientific) as per the manufacturer's instructions. Paired-end libraries were prepared using the Nextera DNA Prep kit, followed by sequencing (2 × 75 bp) on a NextSeq platform (Illumina, Inc., USA). Briefly, Trim Galore v0.6.2 [42] was used to filter the PE reads (*Q* > 30 and length >50 bp). *De novo* assembly and polishing of assemblies were performed using SPAdes v.3.13 [43] and Shovill v1.1.0 [44], respectively. AMR markers were identified using PointFinder [45] and confirmed using Pathogenwatch [46] and Clustal Omega [47]. Whole-genome sequence data are available in DDBJ/ENA/GenBank with the BioProject number PRJNA681740.

### 2.8. Statistical Analysis

The sensitivity, specificity, positive predictive value (PPV), and negative predictive value (NPV) for HRM in comparison with WGS and phenotypic AST were calculated using MedCalc calculator (MedCalc Software bvba, Ostend, Belgium). Sequencing data were used as the reference for the identification of resistance-associated genes and mutations. Phenotypic AST was used as the reference for resistance prediction.

## 3. Results

### 3.1. Genotypic Analysis

A total of 22 paired *N. gonorrhoeae* isolates and clinical specimens and eight WHO control strains were included in this study. Real-time PCR detected the *porA* pseudogene, to identify *N. gonorrhoeae*, in all 22 (100%) clinical isolates and specimens, and eight control strains. We observed that the *bla*_TEM_ plasmid showed 100% concordance in the detection of high-level resistance to penicillin when phenotypic and genotypic results were compared. Whole-genome sequencing confirmed the presence of *tetM* and showed 100% concordance with the phenotypic data.

*N. gonorrhoeae* antimicrobial target genes (*penA*, *porB, ponA*, *gyrA*, *parC*, *mtrR*, *rpsJ*, and 16s rRNA) were investigated for the presence of resistance-associated mutations using HRM. The performance of the HRM assay to detect the presence or absence of mutations in clinical isolates was compared against the WGS data as the gold standard (Table 2). The sensitivity ranged from 38.5%–100%, and specificity ranged from 44.4%–100%. Positive predictive values ranged from 42.9%–100%, and negative predictive values ranged from 44.4%–100%. Concordance between the two methods was ≥83.3% for porB_G120D, porB_G120K, porB_A121G, mtrR_G45D, rpsJ_V57M, gyrA_S91F, gyrA_D95A, parC_S87R, parCS88P, 16S_C1192U, penA_G545S, and penA_mosaic.

The ability of HRM to detect the presence or absence of these resistance-conferring mutations was further evaluated by comparing HRM with phenotypic characteristics (Table 3). For penicillin, the sensitivity ranged from 4.4%–70.8%, specificity ranged from 33.3%–100%, positive predictive values ranged from 33.3%–100%, and negative predictive values ranged from 14.8%–25.0%. For tetracycline, the sensitivity ranged from 10.3%–100%, the specificity for mtrR_G45D was 100%, positive predictive values ranged from 96.4–100%, and negative predictive value for mtrR_G45D was 3.7%. For ciprofloxacin, the sensitivity ranged from 4.4%–100%, specificity was 100%, positive predictive value was 100%, and negative predictive values ranged from 24.1%–100%. Although no resistance to spectinomycin, azithromycin, cefixime, and ceftriaxone was detected in our clinical isolates, the performance of HRM to detect mutations in the control strains was as follows: sensitivity was 100%, specificity ranged from 93.1%–100%, positive predictive values ranged from 33.3%–100%, and negative predictive values were 100%.

HRM was used to detect chromosomal gene mutations, and RTPCR was used to detect plasmid genes. *∗*Only control strains were used for these targets. All isolates in this study were susceptible to spectinomycin, azithromycin, cefixime, and ceftriaxone. PEN = penicillin, TET = tetracycline, CIP = ciprofloxacin, SPT = spectinomycin, AZM = azithromycin, FIX = cefixime, and CRO = ceftriaxone. ^#^CFM MIC for all isolates was <0.016, and the CRO MIC range was <0.002–0.003.

Concordance between molecular detection (real-time PCR and HRM) and resistance phenotype was ≥93% for *bla*_TEM_ (HLR penicillin), rpsJ_V57M (tetracycline), *tetM* (tetracycline), gyrA_S91F (ciprofloxacin), 16SrRNA_C1192U (spectinomycin), mtrR_G45D (azithromycin), penA_D545S (cefixime/ceftriaxone), and penA_mosaic (cefixime/ceftriaxone).

When evaluating the HRM assay to screen the 22 isolates paired clinical specimens (vaginal/urethral swabs) for the presence or absence of resistance-conferring mutations, the concordance of HRM detection between patient isolate and specimen was >90% for all targets (Table 4). These excellent concordance values between patient isolate and paired specimen suggest that the data from Tables 2 and 3 can be inferred to patient specimens.

## 4. Discussion

This study used molecular techniques to identify *N. gonorrhoeae* and to detect genes and mutations associated with antimicrobial resistance. AST revealed that *N. gonorrhoeae* resistance to penicillin, tetracycline, and ciprofloxacin was high in our isolates. We report that spectinomycin, cefixime, and the drugs used in the syndromic management approach, ceftriaxone and azithromycin, remain effective as all isolates were susceptible at the lowest MIC. Our study shows that the sensitivity and specificity of the molecular assays for *bla*_TEM_ (high-level resistance to penicillin), rpsJ_V57M (lower-level resistance to tetracycline), *tetM* (high-level resistance to tetracycline), and gyrA_S91F (resistance to ciprofloxacin) were 100%, with 100% concordance to phenotypic AST data of patient isolates and control strains. Head-to-head comparison of HRM data from isolates and paired clinical specimens showed >90% concordance, which indicates the potential to identify AMR targets directly from *N. gonorrhoeae*-positive vaginal/urethral specimens.

While multiple commercially available NAATs are available to diagnose STIs, this study used real-time PCR to detect the species-specific *porA* pseudogene that identifies *N. gonorrhoeae*. The concordance was 100% when compared with WGS. Other studies have also reported excellent clinical performance of *porA* [12, 27, 30, 48–50]. There have been reports of *porA* mutants which result in a false-negative for identification [51]; however, this was not detected in our isolates. This cost-effective real-time assay, as a diagnostic marker for reliable identification, can be introduced in a clinic management algorithm, and a result can be obtained within two hours compared to culture, which can take up to 48 hours for isolation of *N. gonorrhoeae*.

The prevalence of the *bla*_*TEM*_ (high-level resistance) in our study was 79%. This is similar to a recent study from Africa, which reported a prevalence of 72% [52]. Resistance in isolates with intermediate MICs was chromosomally mediated. Modelling analysis identified *tetM* and rpsJ_V57M to be excellent predictors of resistance to tetracycline [53]. All isolates in this study were resistant to tetracycline. The prevalence of the *tetM* plasmid (high-level resistance) was 95%. This is consistent with that of Kenya and South Africa reported recently as 86% and 92%, respectively [52, 54]. rpsJ_V57M (lower-level resistance) was also detected in all isolates resistant to tetracycline. This is similar to that reported in Vietnam [55], but higher compared to that found in Johannesburg, South Africa (70%) [56], Brazil (39%) [57], and Ukraine (67%) [58].

Resistance to ciprofloxacin was detected in 82% of isolates, and gyrA_S91F SNP was detected in all resistant isolates. Other studies have also shown that this SNP is an excellent target for ciprofloxacin resistance detection [55, 56]. The only available commercial assay for resistance detection, SpeeDX, has been developed based on this mutation.

All isolates from our patient population were susceptible to spectinomycin, and using HRM, we detected the resistance-associated SNP C1192U in the control strain. Spectinomycin resistance is exceedingly rare globally [59], and based on local resistance data, it is an effective treatment for genital and anorectal gonococcal infection [60]. A combination of ceftriaxone and spectinomycin is currently used in Japan [11]. South Korea also effectively treats gonorrhoea with spectinomycin, and resistance has not been reported since 1993 [61]. It is reassuring to know that we have the option to preserve the effectiveness of azithromycin and cefixime/ceftriaxone for use in gonorrhoeal infection by using spectinomycin as first-line therapy.

A limitation of this study is the small sample size, which has resulted in the low performance characteristics of some of the SNP targets when comparing HRM and WGS data. To better determine the performance, further evaluation of these drug targets is required on a much larger sample set from clinical specimens collected from hospitals and clinics across the different provinces in South Africa. *porA* mutants that result in false-negative identification for *N. gonorrhoeae* have been found in other studies [51], and in our subsequent studies, a combination of *porA* and *opa* will be used for identification. Also, the principal gene for azithromycin resistance, 23S rRNA, was not included in this HRM study and will be included in subsequent studies. However, from WGS data analysis, none of the isolates had mutations present in their 23S rRNA genes. At present, due to unknown and novel mechanisms of resistance, genotypic AMR prediction cannot completely replace phenotypic AMR; therefore, continued and updated surveillance of local isolates is needed to identify mutations associated with resistance so that local in-house assays can be updated accordingly.

Cost-effective molecular diagnostic tools are required for rapid AMR detection, especially in low–middle-income countries. Using a diagnostic tool such as HRM, which is affordable and where multiple targets can be run in a single analysis, is an option for use in antimicrobial stewardship, rather than the currently used syndromic management approach. However, a functioning laboratory and trained personnel are required. We show that, in our local setting, the *porA* pseudogene can be used reliably to detect *N. gonorrhoeae*, *bla*_TEM_ can be used to detect HLR to penicillin, *tetM* and rpsJ can be used to detect resistance to tetracycline, and gyrA_S91F can be used to detect resistance to ciprofloxacin.

## Figures and Tables

**Table 1:** Oligonucleotides used in this study.

Target	Primer 5′-3′
*porA*_F	F_CCGTGCGTTACGATTCCCCC
*porA*_R	R_ACAGCCGGAACTGGTTTCATCTG
*bla*_TEM__F	F_ATAGACAGATCGCTGAGATAGGTGC
*bla*_TEM__R	R_AAAAGCGGTTAGAGCGGCTATTG
*tetM*_F	F_CCAGCCCCGTCGTCCAAATAGTC
*tetM*_R	R_GCATCAATCATTTGCTCATGTGGC
*penA*_F	F_CCGTGTGATTGTGGCGGTAACC
*penA*_R	R_TGCCCAAGATGTTCAGGCTGC
G545S	F_GCCGACTGCAAACGGTTACTACA
mosaic	F_GCCGACTGCAAACGGTTACTACG
*ponA*_F	F_GAGCGGTCGATAATGAGAAAATGG
*ponA*_R	R_GCATCCAGCGAAACCAAAGC
L421P	F_GGTGGTTCAAGAGCCGTTGCC
*porB*_F	F_CAACAAACAATCCTTCGTCGGCTTG
*porB*_R	R_GGCAAATTCGGGAGAATCGTAGCG
G120D	F_CAGCCCCCTGAAAAACACCGA
G120K	F_CAGCCCCCTGAAAAACACCA
A121G	F_GGATTCCCAAGCATTGACGTTGCC
A121D	F_GGATTCCCAAGCATTGACGTTGT
*rpsJ*_F	F_GCGTTTCAACATTTTGCGTTCTCC
*rpsJ*_R	R_CATCGGTAGTTTTATCGGTCCAATCC
V57M	F_AACATTTTGCGTTCTCCGCACA
*gyrA*_F	F_AAAATAACTGGAATGCCGCCTAC
*gyrA*_R	R_GAAGTTGCCCTGTCCGTCTATC
S91F	F_TACCACCCCCACGGCGATTT
D95A	F_CGCCATACGGACGATGGTGG
D95G	F_CGCCATACGGACGATGGTGCC
*parC*_F	F_CGTGGTCGGCGAGATTTTGG
*parC*_R	R_CGAACCGAAGTTGCCGATGC
S87R	F_TACCATCCGCACGGCGACC
S88P	F_CATCCGCACGGCGACAGTC
16S rRNA_F	F_AGCCGTAACACAGGTGCTGC
16S rRNA_R	R_GACCATTGTATGACGTGTGAAGCC
C1192U	F_ATAAGGGCCATGAGGACTTGACA
*mtrR*_F	F_GGGTTTCATTATACATACACGATTGC
*mtrR*_R	R_GATGTCGTCGCAGATACGTTGG
G45D	F_TTTGAAATGCCAATAGAGCGCGT

For SNPs, a common reverse primer was used from the chromosomal gene primer set.

**Table 2:** Comparison of high-resolution melt assay and whole-genome sequencing (gold standard) for the detection of antimicrobial resistance determinants in *Neisseria gonorrhoeae* clinical isolates and control strains.

Target	HRM vs. WGS concordance (%)	Sensitivity (95% CI) (%)	Specificity (95% CI) (%)	PPV (95% CI) (%)	NPV (95% CI) (%)
ponA_L421P	66.7	76.2 (52.8–91.8)	44.4 (13.7–78.8)	76.2 (63.0–85.8)	44.4 (21.7–69.7)
porB_G120D	83.3	40.0 (5.3–85.3)	92.0 (74.0–99.0)	50.0 (15.3–84.7)	88.5 (78.8–94.1)
porB_G120K	96.7	75.0 (19.4–99.4)	100 (86.8–100)	100	96.3 (82.7–99.3)
porB_A121G	96.7	75.0 (19.4–99.4)	100 (86.8–100)	100	96.3 (82.7–99.3)
porB_A121D	70.0	85.7 (42.1–99.6)	65.2 (42.7–83.6)	42.9 (28.4–58.6)	93.8 (70.5–98.6)
mtrR_G45D	93.3	66.7 (9.4–99.2)	96.3 (81.03–99.9)	66.7 (19.9–94.1)	96.3 (83.9–99.2)
rpsJ_V57M	93.3	100 (87.6–100)	100 (15.8–100)	100	100
gyrA_S91F	100	100 (85.2–100)	100 (59.0–100)		
gyrA_D95A	96.7	85.7 (42.1–99.6)	100 (85.2–100)	100	95.8 (78.9–99.3)
gyrA_D95G	66.6	38.5 (13.9–68.4)	88.2 (63.6–98, 5)	71.4 (36.5–91.6)	65.2 (54.1–74.9)
parC_S87R	100	100 (2.5–100)	100 (88.1–100)	100	100
parC_S88P	100	100 (15.8–100)	100 (87.7–100)	100	100
16S_C1192U ^*∗*^	100	100 (2.5–100)	100 (88.1–100)	100	100
penA_G545S ^*∗*^	100	100 (2.5–100)	100 (87.7–100)	100	100
penA_mosaic ^*∗*^	100	100 (2.5–100)	100 (88.1–100)	100	100

^*∗*^Only control strains were used for these targets. All isolates in this study were susceptible to spectinomycin, azithromycin, cefixime, and ceftriaxone.

**Table 3:** Performance characteristics of real-time PCR and high-resolution melt assay compared to phenotypic AST (gold standard) to predict antimicrobial resistance in *Neisseria gonorrhoeae* clinical isolates and control strains.

Drug (µg/mL)	Target	Genotypic assay vs. AST concordance (%)	Sensitivity (95% CI) (%)	Specificity (95% CI) (%)	PPV (95% CI) (%)	NPV (95% CI) (%)
PEN (MIC 0.016–.256)	ponA_L421P	63.3	70.8 (48.9–87.4)	33.3 (4.3–77.7)	81.0 (69.5–88.8)	22.2 (7.3–51.0)
	porB_G120D	26.7	12.5 (2.7–32.4)	83.3 (35.9–99.6)	75.0 (27.3–96.0)	19.2 (13.9–26.0)
	porB_G120K	30.0	12.5 (2.7–32.4)	100 (54.1–100)	100	22.2 (19.7–25.0)
	porB_A121G	17.2	4.4 (0.1–22.0)	66.7 (22.3–95.7)	33.3 (5.1–82.2)	15.4 (9.3–24.4)
	porB_A121D	53.3	50.0 (29.1–70.9)	66.7 (22.3–95.7)	85.7 (64.4–95.2)	25.0 (14.3–40.0)
	mtrR_G45D	23.3	11.5 (2.5–30.2)	100 (39.8–100)	100	14.8 (13.2–16.7)
	*blaTEM*	100	100 (81.5–100)	100 (73.5–100)	100	100
TET (MIC 0.75–32)	rpsJ_V57M	96.7	100 (88.1–100)	0 (0–98)	96.7 (96.7–96.7)	
	mtrR_G45D	13.3	10.3 (2.2–27.4)	100 (2.5–100)	100	3.7 (3.3–4.2)
	*tetM*	100	100 (85.2–100)	100 (59.0–100)	100	100
CIP MIC (0.002–2)	gyrA_S91F	100	100 (85.2–100)	100 (59.0–100)		
	gyrA_D95A	43.3	26.1 (10.2–48.4)	100 (59.0–100)	100	29.2 (24.4–34.4)
	gyrA_D95G	46.7	30.4 (13.2–52.9)	100 (59.0–100)	100	30.4 (25.0–36.4)
	parC_S87R	26.7	4.4 (0.1–22.0)	100 (59.0–100)	100	24.1 (22.6–25.8)
	parC_S88P	30	8.7 (1.1–28.0)	100 (59.0–100)	100	25.0 (22.7–27.4)
SPT MIC range (2–31)	16S_C1192U ^*∗*^	100	100 (2.5–100)	100 (88.1–100)	100	100
AZM MIC (0.016–0.38)	mtrR_G45D ^*∗*^	96.7	100 (15.8–100)	96.4 (81.7–99.9)	66.7 (22.6–93.2)	100
FIX/CRO^#^	penA_D545S ^*∗*^	100	100 (2.5–100)	100 (87.7–100)	100	100
	penA_mosaic ^*∗*^	100	100 (2.5–100)	100 (88.1–100)	100	100
	mtrR_G45D ^*∗*^	93.3	100 (2.5–100)	93.1 (73.2–99.2)	33.3 (11.6–65.6)	100

**Table 4:** Evaluation of high-resolution melt assay for the detection of antimicrobial resistance determinants in *Neisseria gonorrhoeae* clinical isolates compared to direct swab specimens.

Target	Isolates (*n* = 22)		Direct specimens (*n* = 22)		Concordance (%)
	Present *n* (%)	Absent *n* (%)	Present *n* (%)	Absent *n* (%)	
ponA_L421P	13/22 (59.1)	9/22 (40.9)	12/22 (54.5)	10/22 (45.5)	95.5
**por_G120D**	4/22 (18.2)	18/22 (81.8)	3/22 (13.6)	19/22 (86.4)	95.5
**porB_G120K**	0/22	22/22 (100)	0/22	22/22 (100)	100
**porB_A121G**	2/22 (9.1)	20/22 (90.1)	2/22 (9.1)	20/22 (90.1)	100
porB_A121D	8/22 (36.4)	14/22 (63.6)	6/22 (27.3)	16/22 (72.7)	90.9
**mtrR_G45D**	0/22	22/22 (100)	0/22	22/22 (100)	100
**rpsJ_V57M**	22/22 (100)	0/22	21/22 (95.5)	1/22 (4.5)	95.5
**gyrA_S91F**	18/22 (81.8)	4/22 (18.2)	18/22 (81.8)	4/22 (18.2)	100
**gyrA_D95A**	6/22 (27.3)	16/22 (72.7)	5/22 (22.7)	17/22 (77.3)	95.5
gyrA_D95G	3/22 (13.6)	19/22 (86.4)	3/22 (13.6)	19/22 (86.4)	100
**parC_S87R**	0/22	22/22 (100)	0/22	22/22 (100)	100
**parC_S88P**	0/22	22/22 (100)	0/22	22/22 (100)	100
**16S_C1192U** ^*∗*^	0/22	22/22 (100)	0/22	22/22 (100)	100
**penA_G545S** ^*∗*^	0/22	22/22 (100)	0/22	22/22 (100)	100
**penA_mosaic** ^*∗*^	0/22	22/22 (100)	0/22	22/22 (100)	100

## Data Availability

Whole-genome sequence data are available in DDBJ/ENA/GenBank with the BioProject number PRJNA681740.
